# Grazing and Supplementation of Dietary Yeast Probiotics Shape the Gut Microbiota and Improve the Immunity of Black Fattening Goats (*Capra hircus*)

**DOI:** 10.3389/fmicb.2021.666837

**Published:** 2021-08-18

**Authors:** Quzhe Emu, Hao Guan, Jiangjiang Zhu, Lin Zhang, Jinsheng Fan, Yang Ji, Yaqiu Lin, Chunmei Li, Xiaobo Dan, Yueda Aguo, Xiaolan Wei, Min Zhang, Bin Zhang, Chao Yang, Bo Li, Chaorui Xiong

**Affiliations:** ^1^Animal Breeding and Genetics Key Laboratory of Sichuan Province, Sichuan Animal Science Academy, Chengdu, China; ^2^Qinghai-Tibetan Plateau Animal Genetic Resource Reservation and Utilization Key Laboratory of Sichuan Province, Southwest Minzu University, Chengdu, China; ^3^Husbandry and Veterinary Technology Promotion Center of Fushun County, Zigong, China; ^4^Rongxian Agricultural Technology Extension Center, Zigong, China

**Keywords:** growth performance, gut microbiota, immune response, feeding modes, goats

## Abstract

This study aimed to investigate the effects of different feeding modes on the growth performance, gut microbiota, and immunity of Black Fattening Goat (*Capra hircus*). A total of 30 goats were grouped in three groups by their feeding modes (pasture grazing group, PG; barn feeding group, BF; barn feeding + probiotics, BF + P; *n* = 10) and the study was performed for 114 days. After a 2-week adaptation period, the first growth performance test was conducted, and the blood and fecal samplings (day 0) were collected on January 17, 2020, while the second and third test and samplings were conducted on days 53 and 100 of feeding. The species-composition of fecal microbiota was analyzed by 16S ribosomal RNA gene-sequencing using PacBio single molecule real time (SMRT) sequencing technology. Both the BF and BF + P groups had the highest (*P* < 0.05) body’s weight and length, and chest circumference at days 53 and 100, especially at day 100, the body’s weight of both the BF groups were more than 18 kg. The levels of immunoglobulin A (IgA) and immunoglobulin G (IgG) were found to be significantly higher (*P* < 0.05) in the PG and BF + P groups at day 100. The PG group exhibited the highest number of operational taxonomic unit (OTUs) and alpha diversity. Firmicutes, Bacteroidetes, and Verrucomicrobia were the predominant phyla in all the fecal samples. The relative abundance of *Akkermansia muciniphila* and *Ruminococcus flavefaciens* were found to be significantly higher (*P* < 0.05) in PG group and BF + P group at day 100, respectively, which might partially explain the significantly higher (*P* < 0.05) levels of IgA and IgG in these two groups. These findings suggested that BF supplemented with 5 g probiotics (*Saccharomyces cerevisiae* and mannan oligosaccharides) per day has the potential to enhance the growth and immunity of Black Fattening Goats.

## Introduction

Gut microbiota has been recognized as an intersection between diet and human health, as highlighted in many studies (Murphy et al., 2010; Gentile and Weir, 2018; Mshelia et al., 2018). There is a symbiotic relationship between the diet and gut microbiota. The diet is considered as one of the most effective regulatory factors for the composition and function of microbiota, while in return, the gut microbiota affects the absorption, metabolism, and storage of nutrients, and exhibits potentially profound effects on the host physiology. Studying the animal gut microbiota, Moeller et al. (2018) reported that the composition of gut microbiota was different among animals. There are extremely significant differences among the diversity and richness of gut microbiota of horses (Mshelia et al., 2018), rats (Rolim et al., 2021), geese (Wang et al., 2020), cows (Uchiyama et al., 2020), and goats (Liu et al., 2020). To date, there have been only few 16S ribosomal RNA (rRNA) gene sequencing technology-based studies, which have investigated the gut microbiota of small ruminants. Huang et al. (2017) investigated the changes in fecal microbiota of lambs fed with purple prairie clover (PPC) and condensed tannins from PPC, and showed that the forage type and condensed tannins had no effects on the composition and α-diversity of fecal microbiota at phylum level. On the other hand, the concentrate supplementation in starch-based diet promoted a simplified rumen microbiota of sheep in terms of network density and richness of bacterial, methanogen and fungal species (Belanche et al., 2019), which indicated that the diet changed sheep’s rumen microbiota. A couple of studies also demonstrated the changes in rumen microbiota during the transition from forage to concentrate diets in cows (Fernando et al., 2010; Zhu et al., 2017). These inconsistent results suggest that the diet affects the ruminant’s gut microbiota, but the degree of effect may depend on the type of diet.

Probiotics are generally proposed as alternatives for sub-therapeutic antibiotics in the livestock industry. The use of yeast and its products as probiotics is well-reported (van der Peet-Schwering et al., 2007; Xu et al., 2018). In many studies, the dietary supplementation of yeast cells and their cultures or cell wall products has been shown to have positive effects on the performance and health of weaned piglets by mitigating the stress and disease-associated negative effects (Li D. et al., 2006), and has also been reported to improve the immunological status of lambs (Ali et al., 2020). However, their mechanism of action in animals is still not clear. Liu et al. (2018) reported that the dietary supplementation of yeast cultures improved the growth and biochemical parameters of grass carps by modulating their gut microbiota. Therefore, we hypothesized that the yeast culture might regulate the gut microbiota of goats, thereby affecting their production performance and immunity.

This study aimed to investigate the effect of different feeding modes [pasture grazing (PG), barn feeding (BF), and barn feeding + probiotics (BF + P)] on the growth performance, gut microbiota, and immunity of Black Fattening Goats. Moreover, the use of a relatively large number of experimental animals and PacBio single molecule real time (SMRT) sequencing allowed the identification of core gut microbiota at species level and generation of correlations between the immune parameters and different species in gut microbiota with different feeding modes.

## Materials and Methods

### Ethics Statement

The animal study was reviewed and approved by the Animal Protection and Utilization Committee of Sichuan Academy of Animal Husbandry Sciences and performed in accordance with Guide to Animal Experiments of Ministry of Science and Technology (Beijing, China). This study did not involve any endangered or protected species.

### Animals and Sample Collection

The study was carried out during January 3, 2020 to April 27, 2020 at Hemushan goat breeding farm, Zigong City, Sichuan Province, China, where the goats were pre-fed for 2 weeks. A total of 30 healthy and genetically unrelated female Black Fattening Goats (*Capra hircus*), having the age of 70 days (8.33 ± 0.55 kg), were randomly allocated into three groups: (1) PG; (2) BF; and (3) BF + P groups. Each group contained 10 goats, divided in five barns, where two goats were fed in a single barn. All the selected goats were treated with the same immune procedure and no serious illnesses was observed prior to the sample collection. The major forage species present in the PG group included *Trifolium repens*, *Elsholtzia splendens, Erigeron canadensis, Lolium perenne*, and *Rheum auriculatum*. The goats in BF group were fed with standard goat diet in the same husbandry. Table 1 enlists the diet ingredients of BF group. The BF + P group was fed with the diet same as that of BF group along with 5 g of probiotics (WeiTaiKang, Sichuan Zhongnong HENGCHUANG Biotechnology Co., Ltd., China) per goat per day. The major active ingredients in probiotics included *Saccharomyces cerevisiae* (activity 1 × 10^5^) and mannan oligosaccharides. The sampling was carried out on different time-points: January 17, 2020 (trail start, day 0), March 10, 2020 (day 53), and April 27, 2020 (day 100). The growth performance (body weight, height and length, and chest circumference) was measured at each time-point. About 2 mL of blood was taken from the jugular vein of goat in EDTA tubes. At the same time, about 20 g of feces was collected and stored in sterile non-enzyme cryopreservation tubes. All the samples were transported to the laboratory in ice within 2 h of collection and stored at −80°C.

**TABLE 1:** Composition of ingredients for Black Fattening Goat feed.

Ingredient	% of total
Corn barn	10
Peanut vine	19
Alfalfa meal	21
Corn	28
Corn germ	9
Bean curd CP43%	4
Expanded soybean	3
Molasses	3
Premix feed	3
Forage to concentrate ratio	1:1

### DNA Extraction

The microbial genomic DNA was extracted from 500 mg of each fecal sample using the PowerSoil/Fecal^®^ DNA Isolation kit (DP812, TIANGEN Biotech Co., Ltd., Beijing, China) following the manufacturer’s instructions. The quality of extracted DNA was observed using 0.8% (w/v) agarose gel electrophoresis and its concentration was measured using a UV-Vis spectrophotometer (NanoDrop 2000, United States). After the total microbial genomic DNA extraction, specific barcode sequencing primers were designed according to the universal primers of 16S rRNA gene: 27F (5′-AGAGTTTGATCCTGGCTCAG-3′) and 1492R (5′-GGTTACCTTGTTACGACTT-3′). The 16S rRNA gene was amplified using PCR. The PCR products were purified, quantified, and homogenized to form a sequencing Library (SMRTbell library). The constructed library was inspected, and the qualified library was sequenced using a PacBio Sequel System. CCS (circular consensus sequencing) reads (SMRT link, version 8.0) were obtained by correcting the original sub-reads. The CCS reads from different samples were identified with barcode sequences using Lima (v1.7.0) software and the chimeras were removed to obtain high quality CCS sequences (1,200–1,650 bps). These sequence data have been submitted to the GenBank databases under accession number PRJNA744356.

### Bioinformatics Analysis

The SILVA database was used to align the resulted sequences. USEARCH (version 10.0) (Edgar, 2013) was used for the clustering of sequences at 97% similarity level. The clustered OTUs (operational taxonomic unit) were then filtered with 0.005% of the number of sequencing columns as threshold. The sequences were rarefied prior to the calculation of alpha diversity indices. In order to determine the diversity and richness of microbiota in the fecal samples, the alpha diversity indices (ACE, Chao1, Shannon, and Simpson indices) were calculated from the rarefied sequences using QIIME2.^1^ PICRUSt2 software was used for the construction of phylogenetic tree and annotation of characteristic sequences, and the IMG microbial genome dataset was used for functional annotation, thereby analyzing the composition of functional genes in different samples in order to predict functional differences among different samples or groups. The *P*-value threshold was 0.05 (*P* < 0.05 meant significant difference). Correlation heat map between taxa abundances and immune indices was constructed by Spearman analysis and screening the data with correlation greater than 0.1. The *P*-value standard of significance was 0.05.

### Immune Indices

Enzyme linked immunosorbent assay (ELISA) diagnostic kit was used for the detection of levels of immunoglobulin A (IgA), immunoglobulin G (IgG), immunoglobulin M (IgM), tumor necrosis factor-α (TNF-α), interleukin-1β (IL-1β), interleukin-6 (IL-6), and interleukin-10 (IL-10) proteins following the manufacturer’s instructions.

### Statistical Analyses

All the analyses were conducted using the general linear model (GLM) procedure of SPSS v22. The data related to growth performance (body weight, height and length, and chest circumference) and immune indices (IgA, IgG, IgM, TNF-α, IL-1β, IL-6, and IL-10) were subjected to two-way repeated measures analysis of variance (ANOVA). The differences among different groups were separated using Bonferroni *post hoc* test, and the *P* < 0.05 was significant.

## Results

### Growth Performance

Table 2 enlists the effect of different feeding modes on the production performance of Black Fattening Goats during the trial window. Through the overall analysis two-way repeated measures ANOVA, it was concluded that different treatments and periods and their interaction had significant effects on the body weight and chest circumference of goats (*P* < 0.05). Periods and their interaction had significant effects on the body height and body length (*P* < 0.05). With the extension of feeding time, the body weight, body height, body length, and chest circumference of goats showed a significant upward trend in the same feeding mode (*P* < 0.05). The body weight and chest circumference of goats in grazing mode were significantly lower than those in BF mode on the days 53 and 100, respectively (*P* < 0.05), while the body weight, body height, body length, and chest circumference of goats in BF + P mode were not significantly different from those in BF mode at the same time point (*P* > 0.05).

**TABLE 2:** Effects of different feeding modes on production performance of Black Fattening Goat (*n* = 10).

Treatment	Period	Body weight (kg)	Body height (cm)	Body length (cm)	Chest circumference (cm)
Grazing (G)	Day 0	8.56 ± 1.58	41.3 ± 2.5	45.5 ± 2.8	48 ± 2.83
	Day 53	10.17 ± 1.66at	44.45 ± 3.06t	46.9 ± 3.48t	50.65 ± 2.65at
	Day 100	13.56 ± 1.94at	50.2 ± 2.86t	52.9 ± 3.25t	58.9 ± 2.96at
Barn feeding (BF)	Day 0	8 ± 1.45	40.9 ± 3.38	44.2 ± 2.7	47.4 ± 2.12
	Day 53	12.63 ± 2.89t	46.7 ± 2.73t	49.6 ± 3.41t	55.6 ± 2.94t
	Day 100	18.06 ± 3.32t	51.91 ± 3t	55.21 ± 4.15t	65.06 ± 2.82t
Barn feeding + probiotics (BF + P)	Day 0	8.44 ± 1.36	40.4 ± 2.22	44.8 ± 3.22	48.3 ± 3.23
	Day 53	14.06 ± 1.33t	47.71 ± 2.3t	53.66 ± 1.92at	57.05 ± 2.11t
	Day 100	18.74 ± 1.91t	53.88 ± 2.48t	55.41 ± 2.5t	64.22 ± 3.66t
Overall analysis	HF	1	1	1	0.985
Treatment	*F*, *P*	7.808, 0.002	1.926, 0.165	3.082, 0.062	8.580, 0.001
Period	*F*, *P*	304.651, <0.001	277.023, <0.001	156.460, <0.001	427.267, <0.001
Interaction	*F*, *P*	13.612, <0.001	4.930, 0.002	8.667, <0.001	10.748, <0.001

*The overall analysis is two-way repeated measures ANOVA. Bonferroni multiple correction was used for the comparison among treatments and periods, the letter “a” indicates the comparison with the barn feeding (BF) group at the same time point (*P* < 0.05), and the letter “t” was used for comparison with day 0 of the same treatment (*P* < 0.05).*

### Immune Indices

The effect of different feeding modes on the immune indices of Black Fattening Goats during the trial window is listed in Table 3. Through the overall analysis two-way repeated measures ANOVA, it was concluded that different periods had significant effects on the Ig(A), Ig(G), Ig(M), TNF-α, IL-1β, IL-6, and IL-10 of goats (*P* < 0.05), while treatments had significant effects on the Ig(A) and Ig(G) of goats (*P* < 0.05). With the extension of feeding time, the Ig(A), Ig(G), and Ig(M) significantly increased (*P* < 0.05), while TNF-α, IL-1β, IL-6, and IL-10 showed a decrease trend in BF + P mode (*P* < 0.05), grazing and BF mode had the same trend as TNF-α, IL-1β, IL-6, and IL-10 concentration. Grazing and BF + P modes had the highest Ig(A) and Ig(G) concentration at day 100 (*P* < 0.05). Ig(M), TNF-α, IL-1β, IL-6, and IL-10 concentration of goats had no significant difference among treatments at the same time point (*P* > 0.05).

**TABLE 3:** Effects of different feeding modes on immune indices of Black Fattening Goat (*n* = 10).

Treatment	Period	Ig(A) (μ g/mL)	Ig(G) (μ g/mL)	Ig(M) (μ g/mL)	TNF-α (pg/mL)	IL-1β (pg/mL)	IL-6 (pg/mL)	IL-10 (pg/mL)
Grazing (G)	Day 0	181.89 ± 20.45a	6.6 ± 0.8a	837.09 ± 193.81	181.63 ± 22.95	70.32 ± 13.75	144.98 ± 22.42	29.4 ± 7.59
	Day 53	190.64 ± 28.47	8.29 ± 0.77at	1,155.78 ± 206.71t	141.49 ± 22.3t	54.67 ± 11.38t	89.99 ± 10.8t	42.57 ± 6.72t
	Day 100	238.09 ± 22.53at	8.92 ± 0.66at	1,409.79 ± 234.9t	107.74 ± 16.25t	31.41 ± 11.69t	77.59 ± 17.1t	50.64 ± 5.88t
Barn feeding (BF)	Day 0	132.21 ± 17.59	5.09 ± 0.52	780.56 ± 142.63	175.51 ± 25.51	74 ± 12.12	133.41 ± 29.71	29.46 ± 6.4
	Day 53	159.96 ± 31.75	7.14 ± 0.6t	1,119.44 ± 116.86t	145.67 ± 24.3t	45.96 ± 7.74t	103.24 ± 25.64t	48.91 ± 9.06t
	Day 100	196.2 ± 25.39t	7.62 ± 1.12t	1,449.73 ± 211.17t	109.02 ± 16.58t	33.52 ± 13.14t	81.27 ± 15.85t	51.49 ± 8.46t
Barn feeding + probiotics (BF + P)	Day 0	160 ± 29.61	6.82 ± 0.99a	790.35 ± 209.57	181.6 ± 14.97	71.62 ± 16.74	141.87 ± 25.81	32.36 ± 4.67
	Day 53	193.43 ± 18.37at	7.72 ± 0.95t	1,172.4 ± 164.32t	149.15 ± 14t	48.09 ± 12.96t	102.48 ± 14.18t	46.39 ± 8.79t
	Day 100	233.29 ± 27.19at	8.98 ± 0.53at	1,464.88 ± 247.67t	100.26 ± 17.93t	27.52 ± 12.88t	74.82 ± 15.34t	54.32 ± 8.02t
Overall analysis	HF	1	1	1	0.978	1	1	0.971
Treatment	*F*, *P*	21.185, <0.001	24.141, <0.001	0.141, 0.869	0.001, 0.999	0.424,0.659	0.071,0.932	1.282,0.295
Period	*F*, *P*	47.069, <0.001	62.157, <0.001	69.238, <0.001	96.699, <0.001	73.963, <0.001	80.239, <0.001	75.866, <0.001
Interaction	*F*, *P*	0.688, 0.604	1.316, 0.286	0.223, 0.924	0.577, 0.680	0.765, 0.553	1.321, 0.275	0.718, 0.584

*The overall analysis is two-way repeated measures ANOVA. Bonferroni multiple correction was used for the comparison among treatments and periods, the letter “a” indicates the comparison with the barn feeding (BF) group at the same time point (*P* < 0.05), and the letter “t” was used for comparison with day 0 of the same treatment (*P* < 0.05).*

### Analysis of DNA Sequencing Data and Microbial Diversity Indices

**FIGURE 1:**
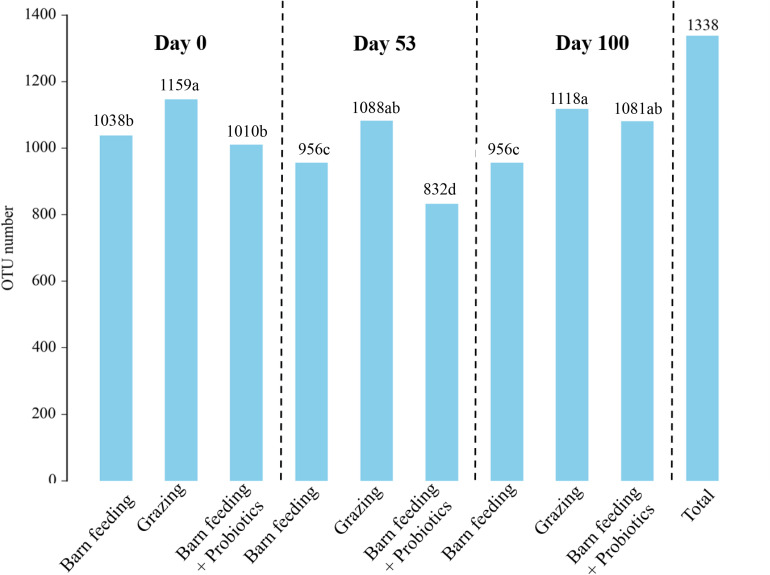
Effects of different feeding modes on the bacterial OTUs in Black Fattening Goat at different growth stages (*n* = 10). ^*a*– *d*^Values with different lowercase letters show significant differences among treatments (*P* < 0.05).

A total of 912,070 CCS reads were obtained from 86 samples. Each sample produced at least 8,699 CCS reads, with an average of 10,605 CCS reads. A total of 1,338 OTUs were identified in all the samples taken at all time-points. The flattened rarefaction curve indicates that the sequencing depth has basically covered all species in the sample (Supplementary Figure 1). In general, the number of OTUs showed a decreasing trend in the beginning and then showed an increasing trend (Figure 1). The number of OTUs in PG group were the highest (*P* < 0.05) in each period, which were over 1,000. The number of OTUs in the BF group first decreased with the increase in feeding duration and then stabilized, while the number of OTUs in BF + P group first decreased to 832 on day 53, and then increased to 1,081 on day 100.

**FIGURE 2:**
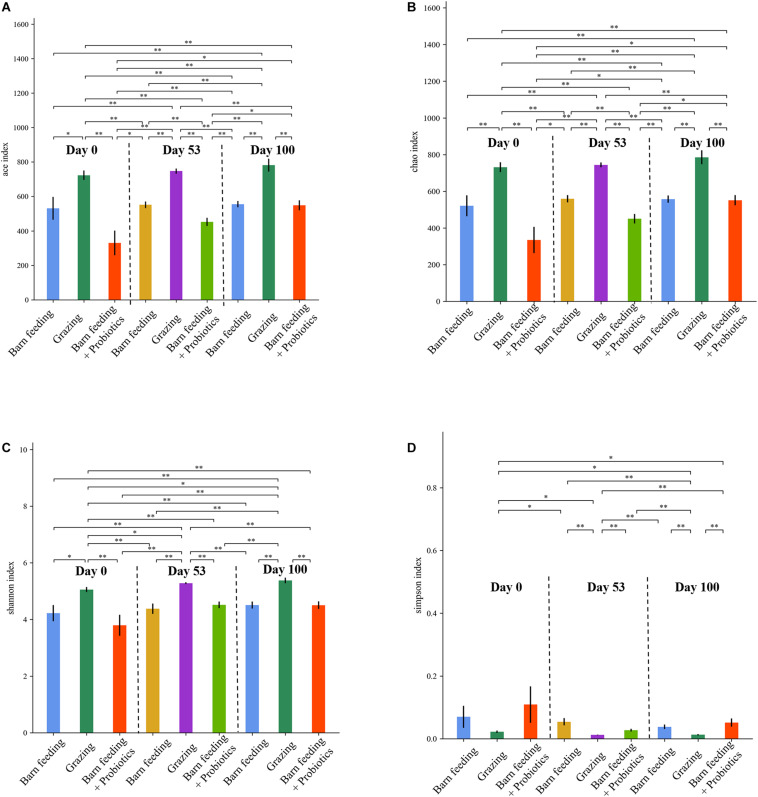
Effects of different feeding modes on the bacterial alpha indexes in Black Fattening Goat at different growth stages (*n* = 10). **(A)** Effects of different feeding modes on the ACE index in Black Fattening Goat at different growth stages. **(B)** Effects of different feeding modes on the Chao1 index in Black Fattening Goat at different growth stages. **(C)** Effects of different feeding modes on the Shannon index in Black Fattening Goat at different growth stages. **(D)** Effects of different feeding modes on the Simpson index in Black Fattening Goat at different growth stages. * show significant differences among treatments (*P* < 0.05), ** show significant differences among treatments (*P* < 0.01).

The ACE, Chao1, Shannon-Wiener, and Simpson indices are the multiple α-diversity indices, which were used to analyze the richness and diversity of microbiota in the fecal samples. The PG group had significantly higher (*P* < 0.05) ACE, Chao1, and Shannon-Wiener indices as compared to the other treatment groups at each time-point (Figure 2). Furthermore, that of PG and BF groups did not show significant changes at each time-point while the BF + P group showed a significant increasing trend (*P* < 0.05). On the contrary, the PG group had the lowest (*P* < 0.05) Simpson index at all the time-points, and showed a decreasing trend with the increase in feeding duration.

### Composition of Microbiota at Different Taxonomical Levels

**FIGURE 3:**
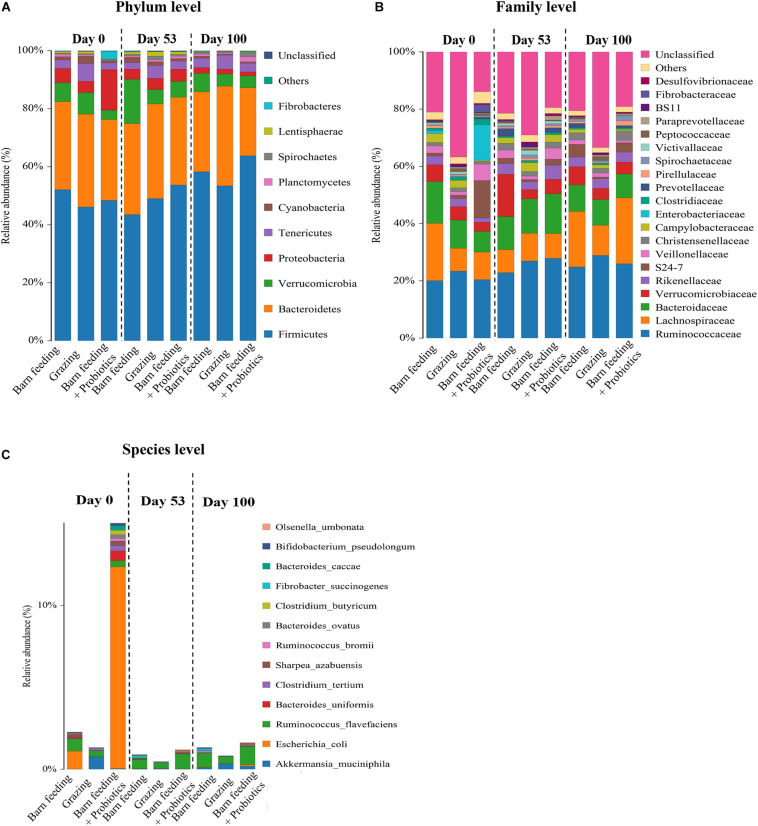
Effects of different feeding modes on the bacterial relative abundance in Black Fattening Goat at different growth stages (*n* = 10). **(A)** Effects of different feeding modes on the bacterial relative abundance at phylum level in Black Fattening Goat at different growth stages. **(B)** Effects of different feeding modes on the bacterial relative abundance at family level in Black Fattening Goat at different growth stages. **(C)** Effects of different feeding modes on the bacterial relative abundance at species level in Black Fattening Goat at different growth stages.

As shown in Figure 3A, at the phylum level, Firmicutes were the most predominant phylum in all the 86 samples, followed by Bacteroidetes, Verrucomicrobia, Proteobacteria, and Tenericutes. With the increase in feeding duration, the Firmicutes showed an increasing trend, while Proteobacteria showed a decreasing trend. The highest relative abundance of Firmicutes were observed in BF + P group at day 100, which exceeded 60% of the total species in fecal microbiota. The PG group showed the highest relative abundance of Bacteroidetes at day 100, which was around 30%. The relative abundance of Proteobacteria decreased significantly (*P* < 0.05) in the BF + P group at day 100 as compared to that at day 0. The relative abundance of Verrucomicrobia in the BF group at day 100 was significantly lower (*P* < 0.05) than that at day 53, but was still higher than the other groups at the same time-point.

A total of 28 families having the relative abundance of more than 0.1% were identified in all fecal samples at family level (Figure 3B). Ruminococcaceae, Lachnospiraceae, and Bacteroidaceae were the most abundant families. About 40% abundance of bacteria could not be classified in the PG group at all the time-points, while the other groups had only about 20% of unclassified bacteria. The PG group had the highest relative abundance of Ruminococcaceae (*P* < 0.05) at day 100, while BF + P group had the highest (*P* < 0.05) abundance of Lachnospiraceae at day 100.

At the species level (Figure 3C), a total of 13 different species, having the abundance of more than 0.1%, were identified in all the fecal samples. Interestingly, the relative abundance of *Escherichia coli* was the highest in the BF + P group at day 0. However, with the increase in feeding duration, the abundance of *E. coli* decreased significantly. The PG group had the highest (*P* < 0.05) abundance of *Akkermansia muciniphila* at day 0. *A. muciniphila* was also observed in the PG and BF + P groups. *Ruminococcus flavefaciens* appeared in all the feeding modes at each time-point. At the late stage of feeding, *R. flavefaciens* occupied the same corresponding position in all the groups.

### Correlation Between Bacteria and Immune Indices

**FIGURE 4:**
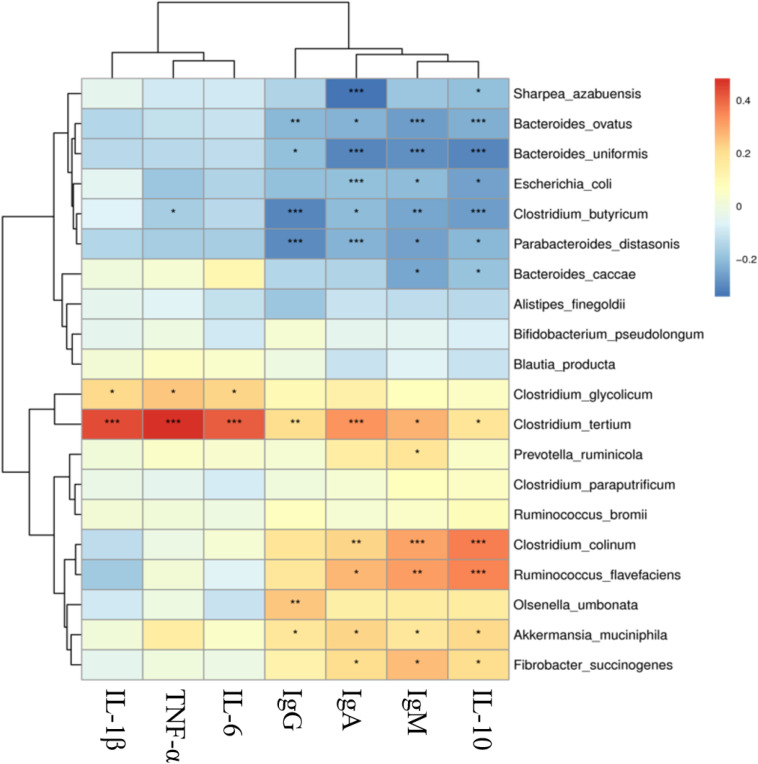
Correlation analysis of bacterial species and immune indexes in Black Fattening Goat. The colors represent different level of the *r* value, and the points represent significance, *P < 0.05, **P < 0.01, ***P < 0.001.

Significant correlations were observed among the fecal microbiota at species level and immune indices (Figure 4). There was a significant (*P* < 0.05) positive correlation between *A. muciniphila* and IgG (0.176), IgM (0.173), IgA (0.223), and IL-10 (0.211). *Fibrobacter succinogenes*, *Clostridium colinum*, and *R. flavefaciens* had significant (*P* < 0.05) positive correlations with IgA, IgM, and IL-10. *Clostridium tertium* had a significant (*P* < 0.05) positive correlation with all the immune indices, especially with the IL-1β (0.440), TNF-α (0.483), and IL-6 (0.414). On the other hand, there were significant (*P* < 0.05) negative correlations among *E. coli* and IgM (−0.203), IgA (−0.199), and IL-10 (−0.252). Besides, *Bacteroides caccae*, *Bacteroides ovatus*, *Clostridium butyricum*, and *Parabacteroides distasonis* were significantly (*P* < 0.05) negatively correlated with IgG, IgM, IgA, and IL-10.

## Discussion

### Goat Fecal Microbiota

To the best of our knowledge, this is the first study of its type to characterize the fecal microbiota of goats in different feeding modes using PacBio SMRT sequencing of 16S rRNA gene. The present study identified a total of 10 different phyla in all the fecal samples. Firmicutes and Bacteroidetes were the top two most predominant phyla, respectively, which accounted for more than 70% of total sequences. The predominance of these two phyla has been previously reported in the fecal microbiota of different mammalian species, including cattle (Kim et al., 2015), humans (Ley et al., 2008), and pigs (Holman and Chénier, 2014), as well as in the rumen of lambs (Morgavi et al., 2015). These findings indicated the importance of these phyla in the ecosystem of gut microbiota. As compared to other studies (Huang et al., 2017; Wang et al., 2018), which reported the relative abundances of Firmicutes and Bacteroidetes in lamb and goat fecal samples to be more than 90%, these two phyla were shown in this study to be found in relatively low abundance (around 70%). We found that Verrucomicrobia was the third most abundant bacterial phylum in goat fecal samples, which accounted for at least 5% of the total microbiota in each group. This finding was not consistent with the previous study, which reported its abundance to be not more than 2% in lamb feces (Huang et al., 2017). Verrucomicrobia is commonly found in fresh water (Chiang et al., 2018) and soil (Bergmann et al., 2011). The most common member of phylum Verrucomicrobia, which is found in human gut, is *Akkermansia* that degrades the excess mucin produced by the intestinal wall (Derrien et al., 2008). These differences might be related to the breeds and environmental conditions of goats.

A total of 13 species were identified from all the fecal samples, where each species had individual abundance of more than 0.1%. *Akkermansia*, the mucin-degrading bacteria, has been reported to be closely associated with immunity (Ganesh et al., 2013), obesity, and type 2 diabetes of hosts (Plovier et al., 2016). The increasing abundance of *A. muciniphila* can lower the body fat mass, improve glucose homeostasis, and decrease the inflammation of adipose tissues, as reported in both the mice (Ganesh et al., 2013) and human (Shin et al., 2014) studies. The abundance of *A. muciniphila* was observed in the PG and BF + P groups to be significantly higher at day 100, while these two groups also had the highest levels of IgA and IgG, which indicated that the high abundance of *A. muciniphila* might be correlated with the high levels of IgA and IgG, thereby improving the immunity of goats. The correlational analysis of bacteria with the immune indices, showing a significant positive correlation among *A. muciniphila* and IgG (0.176), IgM (0.173), IgA (0.223), and IL-10 (0.211), also supported this observation. The incidence of diarrhea in goats is extremely high, which gradually improves with age. *E. coli* has been shown to be closely associated with diarrhea (Türkyllmaz et al., 2014). In the current study, *E. coli* was mainly found in both the BF groups at day 0, especially in the BF + P group, where its abundance exceeded 10%. However, with the increase in feeding duration, its abundance decreased significantly. *R. flavefaciens*, major cellulose-degrading bacteria, can produce a large amount of cellulase and hemicellulose (Flint et al., 1989; Kirby et al., 1997), which are mainly xylanase. *R. flavefaciens*, a cellulolytic bacterium, is usually found in rumen of cattle (Leng et al., 2011) and lambs (Chun-Tao et al., 2016). Due to the limitations in sequencing technologies, only genus *Ruminococcus* has been reported in the fecal samples of lambs (Huang et al., 2017) and goats (Wang et al., 2018). The current study showed that the *R. flavefaciens* was predominant species in all the feeding groups at day 53, which indicated that the ability of the goats to digest and degrade fibers increased with age. Interestingly, the abundance of *R. flavefaciens* in the BF group at days 53 and 100 was found to be significantly higher than that in the PG group. This might further explain the lower body weights of goats in the PG group than that of the BF groups, which may be attributed to the lower abundance of fiber-degrading bacteria in their intestines. *Clostridium* has long been thought to be closely correlated with diarrhea and intestinal toxemia in ruminants, and its toxins affect body through different pathways (Lewis and Naylor, 1998). *Clostridium tertium* has been reported to be associated with spontaneous peritonitis (Butler and Pitt, 1982). A significant decrease in the abundance of *Clostridium tertium* was observed in all the feeding groups at day 53 of feeding, suggesting a decrease in its abundance in the goats’ intestinal tract with the increase in their age, which was more conducive to the host’s health.

### Effects of Feeding Modes on Fecal Microbiota and Growth Performance

By measuring the growth performance of all the feeding groups of goats, we found that the body’s weight, height and length, and chest circumference of the BF and BF + P groups were significantly higher than that of the PG groups at days 53 and 100. The growth of PG groups was slow, whereas some goats even did not grow from day 0 to day 53. The changes of feeding pattens affect the feeding habits and nutrient metabolism of animals, and then affect the deposition of protein and fat (Zarrinpar et al., 2014). In current study, the lowest growth rate and final body weight of PG group may be due to the change of feeding mode, which changed the feeding habits of lambs. They need more activities and consume more energy to get enough food. Besides, based on the analysis of gut microbiota, we found a significantly lower abundance of Firmicutes and higher abundance of Bacteroidetes in the PG group at days 0 and 100. The members of Firmicutes contain genes that are related to the energy metabolism and decomposition of substances, such as fiber and cellulose (Kaakoush, 2015), and the members of Bacteroidetes degrade proteins and carbohydrates (Waite and Taylor, 2014). Therefore, this study suggested that the gut microbiota of PG group had poor abilities related to the energy metabolism and decomposition of substances, such as fiber and cellulose. This poor ability to obtain energy might be one of the reasons for their poor growth performance. In addition, the BF and BF + P groups were found to have higher Firmicutes/Bacteroidetes ratio. The high ratio of these two phyla is beneficial to animals for gut microbiota-mediated energy harvesting (Huan et al., 2016), which assists the host to maintain a metabolic balance and obtain better growth performance (Ley et al., 2006; Murphy et al., 2010).

### Effect of Feeding Modes on Fecal Microbiota and Immunity

The current study found that the number of OTUs were the highest in PG group from day 0 to day 100, which suggested a greater diversity of fecal microbiota in PG group. The highest ACE, Chao1, and Shannon-Wiener indices in PG group also indicated a greater species diversity. The shift from BF to grazing diet caused an increase in the bacterial numbers and diversity, which has been suggested as an adaptation strategy to digest the forage diets (Belanche et al., 2012). In this dietary situation, the structure of bacterial communities was positively correlated with the high microbial complexity-associated parameters, such as the protozoan concentration or diversities of bacteria, fungi, and methanogens. A similar correlational pattern was observed for methanogens and fungal communities, suggesting that the adaptation of rumen microbiota to degrade the fresh pasture-grazing diets indicated a larger number of microbes working together than to degrade the barn diets (McAllister et al., 1994). A couple of studies have demonstrated that the ratio of Firmicutes/Bacteroidetes and diversity of microbiota decreased during the transition from high forage to high grain diets (Fernando et al., 2010; Tapio et al., 2017). These observations were consistent with our results. Le Chatelier et al. (2013) reported that the increased consumption of high-fiber diets, such as fruits and vegetables, increased the diversity of gut microbiota in human, thereby improving some clinical symptoms associated with obesity. Consistent with these previous observations, this study indicated significantly higher levels of IgG and IgA in the PG group with increased diversity and abundance of fecal microbiota, suggesting that in order to adapt to more complex and high fiber food sources in grazing grassland, the intestinal microbiota of goats became more diverse, which might enhance their immunity.

Yeast extracts contain various bioactive compounds, including nucleotides and cell wall’s polysaccharides (specifically β-glucan and α-mannan), which are considered as beneficial for the growth and gut-health of animals, including piglets (Davis et al., 2004), dairy cow (Kowalik et al., 2012), and lambs (Wojcik, 2010). The supplementation of yeast extracts has been shown to exhibit an immuno-stimulating effect, which is associated with the β-glucans and α-mannans present in yeast’s cell wall. For instance, it has been suggested that the β-glucans of yeast could prevent the elevation of pro-inflammatory cytokines while enhancing the production of anti-inflammatory cytokines when piglet’s immune system was stimulated with lipopolysaccharide (Li et al., 2005) or *E. coli* (Li D.F. et al., 2006). The supplementation of yeast-derived α-mannans in piglet’s diets had been reported to be associated with the improved weight gain and feed efficiency, and enhanced function of macrophages in the lamina propria of intestine (Davis et al., 2004). The current study showed significantly higher levels of IgG and IgA in the BF + P group as compared to the BF group at days 53 and 100, which suggested that the dietary supplementation of yeast extracts improved goats’ immune system. The dietary supplementation of yeast extracts has been proved to positively transform the composition of gut microbiota in piglets. For instance, Mateo (2005) reported high abundance of beneficial *Lactobacillus* and *Bifidobacteria* and lower abundance of *Clostridium perfringens* in the fecal samples of piglets that were fed with yeast extract-supplemented diets. The compositions of microbiota were compared between the BF and BF + P groups at days 0, 53, and 100. The species richness, diversity, and evenness of microbiota did not show significant differences between these groups, suggesting that the alpha diversities were not affected significantly by the supplementation of yeast’s probiotics during the experimental period of this study. The alpha diversities of gut microbiota has been reported to increase with aging in pigs (Casellas et al., 2014; Frese et al., 2015). In the current study, we also found that the alpha diversities increased gradually from day 0 to day 53, but then plateaued after day 53 to day 100, which was consistent with the observation in pigs by Frese et al. (2015).

## Conclusion

The gut microbiota of Black Fattening Goat can be altered by modifying their diet, which affects their immunity. Firmicutes, Bacteroidetes, and Verrucomicrobia were the predominant phyla in all the fecal samples. The BF benefited the growth performance of goats, while PB and BF + P improved their immunity by positively modulating the proliferation of beneficial gut microbiota, such as *A. muciniphila* and *R. flavefaciens*. These findings suggested that BF supplemented with 5 g probiotics (*S. cerevisiae* and mannan oligosaccharides) per day has the potential to enhance the growth and immunity of Black Fattening Goats.

## Data Availability Statement

The datasets presented in this study can be found in online repositories. The names of the repository/repositories and accession number(s) can be found in the article/ Supplementary Material.

## Ethics Statement

The animal study was reviewed and approved by the Animal Protection and Utilization Committee of Sichuan Academy of Animal Husbandry Sciences (Permit number: 20191201).

## Author Contributions

QE, HG, JZ, and CX designed the research. QE, LZ, JF, YJ, YL, CL, XD, YA, XW, MZ, BZ, and BL performed the experiments. HG analyzed the data. QE, HG, and JZ wrote the manuscript. All authors contributed to the article and approved the submitted version.

## Conflict of Interest

The authors declare that the research was conducted in the absence of any commercial or financial relationships that could be construed as a potential conflict of interest.

## Publisher’s Note

All claims expressed in this article are solely those of the authors and do not necessarily represent those of their affiliated organizations, or those of the publisher, the editors and the reviewers. Any product that may be evaluated in this article, or claim that may be made by its manufacturer, is not guaranteed or endorsed by the publisher.

## Footnotes

1
https://qiime2.org/

## Supplementary Material

Supplementary Figure 1 Function prediction of gut microbiota in Black Fattening Goat.

Supplementary Figure 2 Interaction network of gut microbiota in Black Fattening Goat.

Supplementary Figure 3 Rarefaction curve of gut microbiota in Black Fattening Goat.
