# Supplementary figures and images for: Grazing and Supplementation of Dietary Yeast Probiotics Shape the Gut Microbiota and Improve the Immunity of Black Fattening Goats (Capra hircus)
Source: Front Microbiol. 2021 Aug 18;12:666837. doi: 10.3389/fmicb.2021.666837 (PMC8416523; doi:10.3389/fmicb.2021.666837)

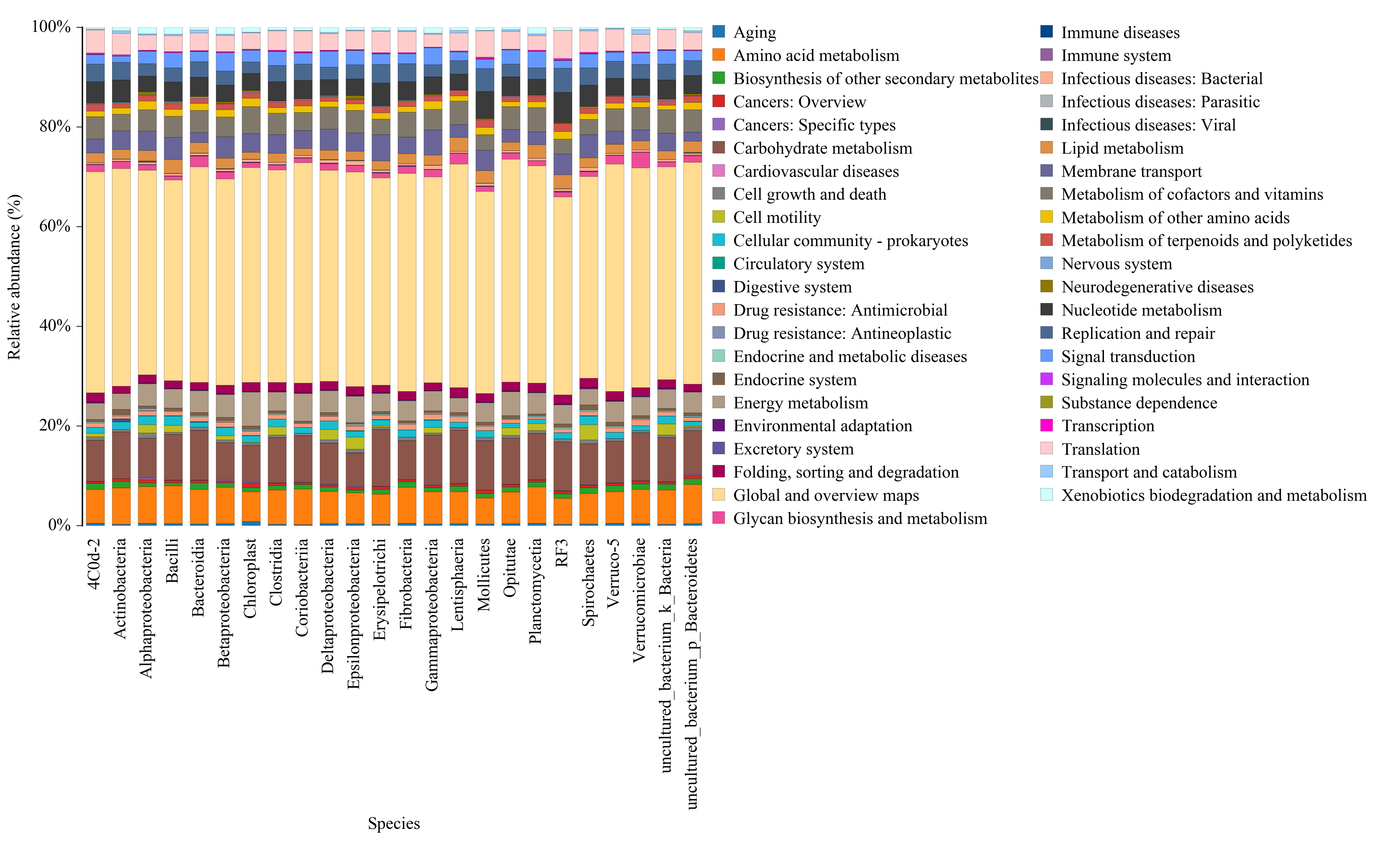

Supplement: Supplementary Figure 1 — Function prediction of gut microbiota in Black Fattening Goat. [file Image_1.png]

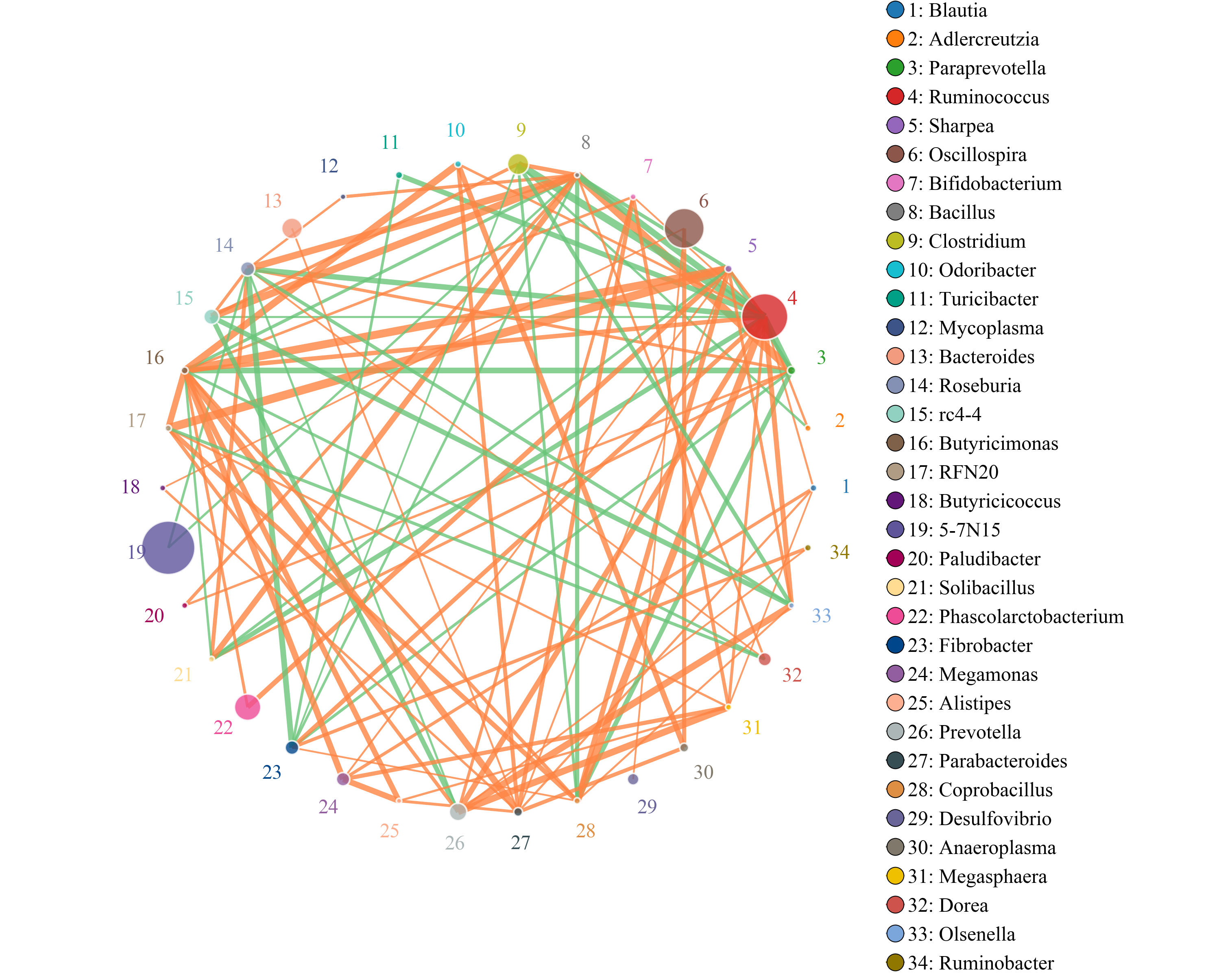

Supplement: Supplementary Figure 2 — Interaction network of gut microbiota in Black Fattening Goat. [file Image_2.png]

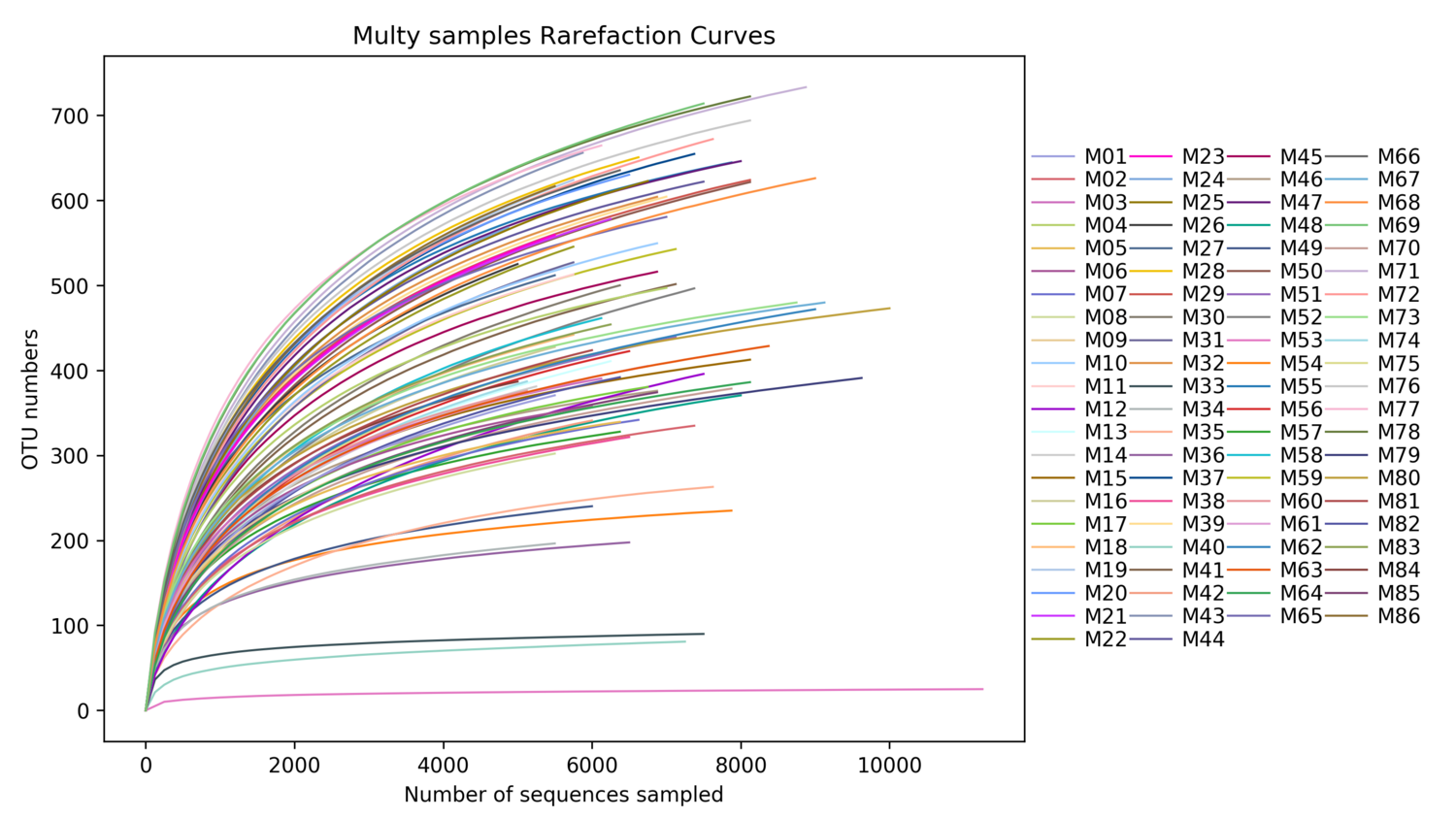

Supplement: Supplementary Figure 3 — Rarefaction curve of gut microbiota in Black Fattening Goat. [file Image_3.png]
